# Synchronous and metachronous melanomas diagnosed at early stages in a patient with dysplastic nevus syndrome^☆^

**DOI:** 10.1016/j.abd.2021.12.011

**Published:** 2023-03-13

**Authors:** Marcelo Moreno, Maria Luiza Mukai Franciosi, Lucas Faria Abrahão-Machado

**Affiliations:** a Faculty of Medicine, Universidade Federal da Fronteira Sul, Chapecó, SC, Brazil; b Pathology Consultancy, Private Practice, Botucatu, SP, Brazil

Dear Editor,

A 34-year-old male patient, phototype II, according to the Fitzpatrick classification, with clinically dysplastic multiple nevi, was surgically treated in 2019 for a superficial spreading cutaneous melanoma with 0.45mm Breslow's thickness, on the lumbar region. He had no family history of melanoma and was referred for photo-dermoscopy/total body mapping (TBM) with the inclusion of all pigmented lesions. For that purpose, macroscopic photos of the patient's skin surface were taken in standard positions. A Canon EOS T7i® camera with EF 50 mm lens (f/1.4 USM aperture) was used to obtain the photos. The camera was attached to the set of hardware that constitutes the Fotofinder Dermoscope 1000®. The images were generated using Medicam full HD 1000®, with non-polarized light, and aqueous gel was used on the interface between the skin and the lens. Afterwards, all lesions were analyzed considering the qualitative method of “pattern analysis”.

A total of 645 melanocytic lesions were documented. Of these, nine showed dermoscopic findings with indication for additional histopathology. Suspicious melanocytic lesions were submitted to excision and three new melanomas were diagnosed: 1) A superficial spreading melanoma, with 0.5mm Breslow's thickness, on the right hypochondrium region (Fig. 1); *in situ* melanoma on the left flank (Fig. 2) and an *in situ* melanoma on the left leg (Fig. 3). The other six lesions were diagnosed as dysplastic nevi (marked dysplasia).

**Figure 1:**
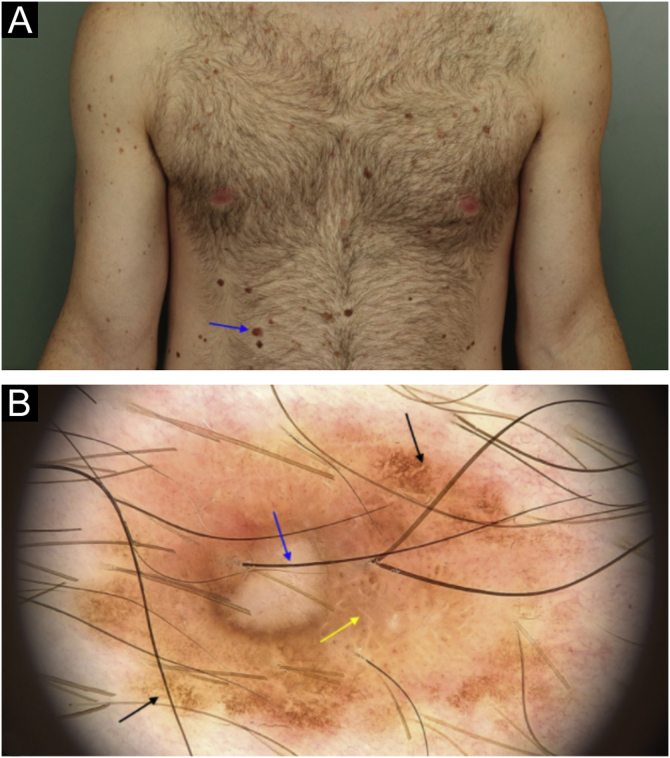
A superficial spreading melanoma, with 0.5mm Breslow’s thickness on the right hypochondrium region: (a) Clinical appearance and location of the lesion (arrow). (b) Dermoscopy showing areas of atypical pigmented network (black arrow), area with reticulated hypopigmentation (yellow arrow); and depigmented/amorphous central area with intermingled linear and irregular vessels (blue arrow).

**Figure 2:**
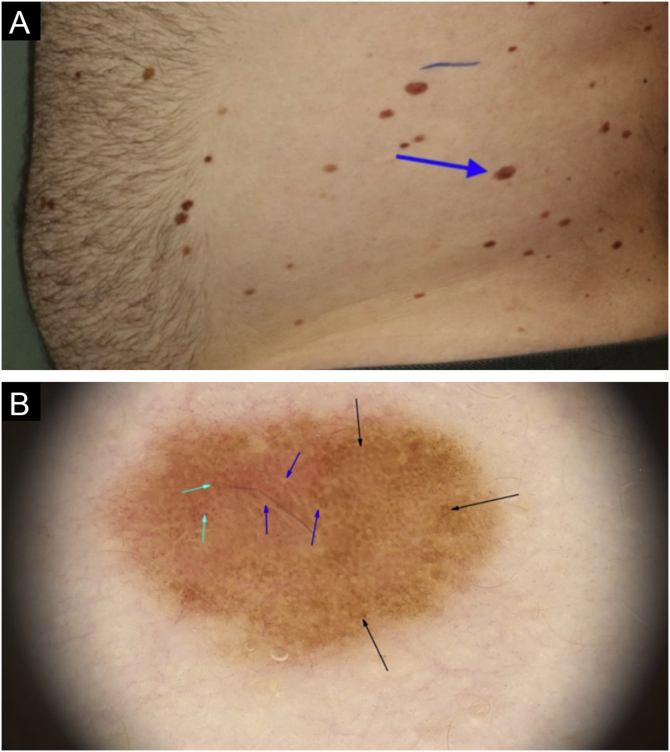
*In situ* melanoma on the left flank: (a) Clinical aspect of the melanoma, showing characteristics similar to other adjacent lesions (blue arrow). (b) On dermoscopy, an irregular pigmented network can be observed (black arrows), associated with reticulated hypopigmentation or inverted network (blue arrow); and peripheral punctiform blood vessels (green arrow).

**Figure 3:**
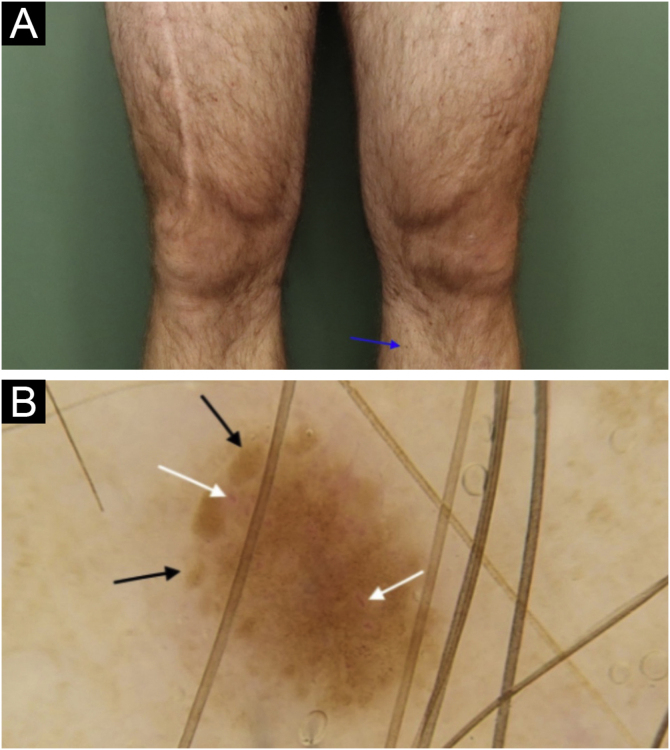
*In situ* melanoma on the anteromedial region of the left lower limb: (a) Clinical aspect of lesion measuring 1.5 mm (blue arrow), with light brown color, similar to other pigmented lesions on both lower limbs. (b) Dermoscopy of the lesion, showing a somewhat symmetrical lesion, light brown in color, containing peripheral globules with irregular distribution and sizes (black arrow); linear, comma shaped and intermingled globular shaped vessels (white arrow).

On histopathology, the atypical nevi showed components of junctional and dermal melanocytic cells (compound nevi) with architectural disarray and asymmetry, in addition to cytological atypia and subepidermal fibroplasia. In the lesions diagnosed as *in situ* melanoma (flank and left leg), in addition to more intense cytological atypia, significant pagetoid dissemination was observed; however, without dermal invasion. The superficial spreading melanoma (abdominal region) showed intense cytological atypia, characterized by pleomorphism and nuclear hyperchromasia, exuberant pagetoid dissemination, and dermal invasion foci with atypical mitotic figures. Immunohistochemistry showed HMB45 positivity in neoplastic cells in both the epidermal and dermal components, indicating the absence of maturation and enhancing the invasive component, unlike what is seen in benign melanocytic lesions (Fig. 4).

**Figure 4:**
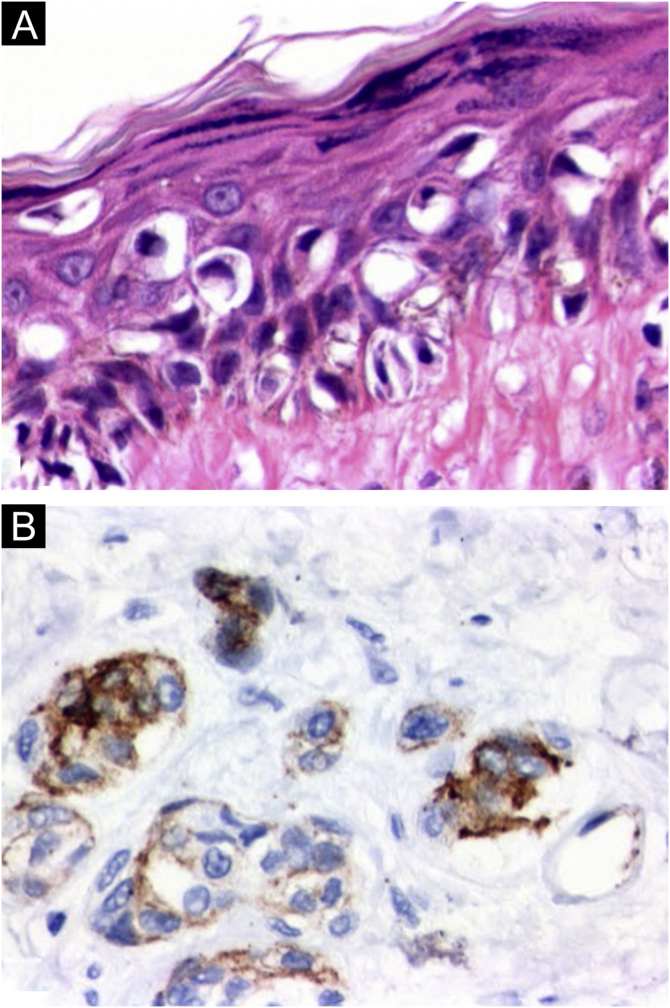
Histopathology: (a) *In situ* melanoma on the left leg showing intense cytological atypia and exuberant pagetoid dissemination (Hematoxylin & eosin, ×100). (b) HMB45 immunohistochemical positivity in neoplastic cells in both the epidermal and dermal components of the invasive melanoma on the abdominal region (×100).

## Discussion

To consider the diagnosis of dysplastic nevus syndrome (DNS) in an individual, a personal or family history of melanoma (in at least one first- or second-degree relative) is necessary, associated with the presence of many nevi; some of them clinically atypical (more than 100 nevi, with at least one nevus measuring more than 8 mm, or one clinically atypical nevus larger than 5 mm).^1^, ^2^ The patient described here had a previous personal history of melanoma, in addition to clinical criteria for the diagnosis of DNS. People with DNS are at high risk for developing cutaneous melanoma (up to 150-fold higher than the general population).^2^, ^3^

Performing the TBM with digital photo-dermoscopy in patients with DNS is an important strategy for the early diagnosis of melanoma and, consequently, the reduction of mortality associated with this neoplasm.^3^, ^4^, ^5^ In this population, approximately 34% to 61% of melanomas are detected exclusively by follow-up with TBM.^6^, ^7^

There is no consensus on how to perform TBM, and all identified pigmented lesions may be included, or only those with clinical and/or dermoscopic suspicion, previously selected according to the ABCD rule criteria or by alterations detected in the initial dermoscopic exam. Including all lesions in TBM requires more time for the acquisition and dermoscopic analysis of the images, but it can increase accuracy, mainly in lesions without clinical suspicion and the ones that do not show characteristics requiring complementary histopathology.^8^, ^9^ At the same time, by analyzing the dermoscopic patterns of all lesions, it is possible to select, with more stringency, the ones that should be included in the subsequent follow-up assessment, in addition to defining the best period for the next examination (three months, six months or 12 months).^9^ In the reported clinical case, although the patient had no family history of melanoma, he had more than 600 melanocytic lesions, several of which with clinical and dermoscopic characteristics of dysplastic nevi (in addition to a previous personal history of melanoma). Diagnosis of the two *in situ* melanomas (mainly the one located on the left leg) was only possible due to the criterion of including all pigmented skin lesions in the initial digital documentation.

## Financial support

None declared.

## Authors' contributions

Marcelo Moreno: Design and planning of the study; data collection, or data analysis and interpretation; drafting and editing of the manuscript or critical review of important intellectual content; collection, analysis, and interpretation of data; effective participation in research orientation; intellectual participation in the propaedeutic and/or therapeutic conduct of the studied cases; critical review of the literature; approval of the final version of the manuscript.

Maria Luiza Mukai Franciosi: Drafting and editing of the manuscript or critical review of important intellectual content; critical review of the literature.

Lucas Faria Abrahão-Machado: Drafting and editing of the manuscript or critical review of important intellectual content; collection, analysis, and interpretation of data; intellectual participation in the propaedeutic and/or therapeutic conduct of the studied cases; approval of the final version of the manuscript.

## Conflicts of interest

None declared.
